# Impacts of Soil Properties and Microbial Community on Fruit Quality and Yield in Ponkan Orchards with Different Comprehensive Performance

**DOI:** 10.3390/plants15050819

**Published:** 2026-03-07

**Authors:** Jiacheng Zhang, Zhijiao Tian, Fei Zheng, Conghui Lu, Xiaochuan Ma, Yuan Yu, Ping Wang

**Affiliations:** The Key Laboratory of the Ministry of Education for Genetics, Breeding and Multiple Utilization of Crops, Institute of Horticultural Plant Genetics and Breeding, College of Horticulture, Fujian Agriculture and Forestry University, Fuzhou 350002, China

**Keywords:** ponkan, soil fertility, microbial community, photosynthetic performance, fruit quality

## Abstract

Fruit quality and yield of citrus orchards are co-regulated by complex interactions among soil properties, microbial communities, and plant physiological processes. However, systematic studies that integrate the soil–microbe–plant–fruit continuum remain limited. This study selected four representative ponkan orchards based on yield and fruit quality performance, and systematically determined and correlated key indicators in the soil–plant–fruit continuum. The results showed that the orchards with higher comprehensive performance exhibited more suitable soil pH, higher contents of soil organic matter and available nutrients, as well as higher activities of soil enzymes including urease and acid phosphatase. Compared with the orchards with lower comprehensive performance, soil bacterial and fungal Chao1, Shannon, and Simpson indices were higher in the orchards with higher comprehensive performance. Among the dominant phyla, the relative abundance of Proteobacteria was significantly higher, while that of Actinobacteria was significantly lower. Leaf photosynthetic indexes (chlorophyll content, net photosynthetic rate, Rubisco activity) of the higher-performing orchards were also significantly higher. Correlation analysis showed that soil microbial diversity and Proteobacteria were significantly positively correlated with soil nutrients, enzyme activities, leaf photosynthesis, fruit quality and yield, while Actinobacteria showed the opposite trend. These results provide a theoretical basis for soil management and high-quality cultivation of ponkan orchards.

## 1. Introduction

Ponkan (*Citrus reticulata* Blanco) is a renowned citrus cultivar widely cultivated in southern China. The fruit is characterized by easy peeling, rich sweet flavor and early maturity, which makes it highly favored by the market [1]. However, the stability of ponkan yield and quality is often restricted by the complex orchard ecological environment. Among the various influencing factors, the soil system serves as the fundamental medium for fruit tree growth, and also acts as a core carrier for maintaining tree health and determining fruit growth and development, playing a fundamental regulatory role [2,3].

Soil fertility is not only related to the ability of soil to supply essential elements for plants, but also involved in the function of maintaining the balance of the entire ecosystem. Soil fertility status is closely associated with soil nutrient supply and microbial community structure, thereby regulating the physiological metabolism of fruit trees and the formation of fruit quality traits, e.g., soluble solids content, titratable acidity. This highlights the crucial role of soil fertility in determining the productivity and fruit quality of ponkan orchards. The soil system is a complex ecological system coupled by abiotic and biotic components. Abiotic components lay the material foundation for fruit development by regulating nutrient bioavailability and the rhizosphere microenvironment for root growth, thus affecting the absorption and utilization of mineral elements by fruit trees. Specifically, soil pH can regulate the absorption of key mineral elements such as calcium, magnesium, iron and zinc by fruit trees through altering nutrient occurrence forms and metal ion toxicity, thereby indirectly affecting fruit quality formation [4,5]. Soil organic matter can not only gradually decompose and release nutrient elements such as nitrogen, phosphorus and potassium, but also has multiple functions including stabilizing soil aggregate structure, improving water and fertilizer retention capacity, promoting root growth, providing energy substrates for soil microorganisms and chelating toxic substances. It can synergistically promote fruit tree growth and improve fruit quality and yield through multiple pathways [6,7,8]. As essential macronutrients for crop growth, nitrogen, phosphorus and potassium are involved in the synthesis of photosynthetic pigments, provide energy for fruit cell division and sugar transport, and affect fruit-set, cell expansion and ripening processes by regulating endogenous hormone levels. Meanwhile, these three elements can maintain cell osmotic pressure and stomatal aperture, ensure tree water supply and photosynthetic efficiency, and ultimately regulate fruit quality in multiple dimensions [9,10].

Numerous studies have demonstrated that soil fertility is a key driver of citrus growth and fruit quality, as it modulates the availability of essential nutrients (e.g., nitrogen, phosphorus, potassium) and shapes the soil microbial ecosystem [11,12]. At the same time, soil microbial communities, as the core executors of soil system functions, also play an important regulatory role in fruit quality. Soil microorganisms participate in soil nutrient cycling and organic matter decomposition, further affecting nutrient uptake by fruit trees [13]. Existing studies have confirmed that strawberry plant growth-promoting rhizobacteria can induce plants to produce volatile substances, promote nitrogen-fixing bacteria colonization and proliferation, and then improve strawberry fruit quality [14]. Soil endophytes have the potential for terpene synthesis, which can directly increase the monoterpene content in the peel of *Citrus reticulata* ‘Chachi’ by providing monoterpene precursors, thereby improving fruit flavor quality [11]. Arbuscular mycorrhizal fungi can form a symbiotic system with strawberry roots, promote root growth and nutrient absorption, and significantly increase the accumulation of anthocyanins in fruits [15]. Soil enzyme activity is the core link connecting the functions of abiotic and biotic components in the soil. It can directly catalyze the biochemical transformation and release process of nutrients such as organic nitrogen, phosphorus and carbon in the soil, and is a key biological indicator for characterizing soil biological activity, fertility level and nutrient supply potential [16,17]. In summary, soil abiotic factors, microbial communities and enzyme activities interact with each other to form a dynamic regulatory network, which ultimately determines the distribution efficiency of photosynthetic assimilates to fruits and fruit quality traits by directly or indirectly affecting tree photosynthetic performance [18,19].

Although there have been some reports on the correlations among soil physical and chemical properties, enzyme activity, microbial communities, tree photosynthetic physiology and citrus fruit quality, most existing studies focus on the isolated analysis of single or a few links [20,21]. Moreover, most relevant research has centered on the individual effects of soil nutrients or microbial communities on citrus growth, with limited systematic exploration of the intricate relationships among soil fertility, microbial community structure, plant physiological traits, and fruit quality. Specifically, the regulatory mechanisms by which soil fertility influences ponkan growth and fruit quality through mediating microbial communities remain unclear, especially in the context of ponkan orchards exhibiting contrasting fruit quality traits. For citrus-producing regions, there is still a lack of integrated, full-chain analysis and mechanistic clarification that incorporates from soil physicochemical properties and enzyme activity to microbial community function, leaf photosynthetic physiology, and ultimately fruit quality into a unified research framework. This leads to difficulties in constructing a precise nutrient management technical system based on soil–microbial synergistic regulation in production practice, and thus fails to achieve the synergistic improvement in fruit yield and quality.

To address the aforementioned research gaps, we proposed the following hypotheses: (1) Soils from orchards with different comprehensive performance levels possess distinct physicochemical properties and microbial community structures, with the orchards with higher comprehensive performance exhibiting superior soil fertility, higher microbial diversity, and enhanced ecological functions; (2) The relative abundance of major bacterial phyla involved in soil carbon, nitrogen, and phosphorus cycling (e.g., Proteobacteria) may be correlated with improved leaf photosynthetic performance and enhanced fruit quality; (3) A close correlation network exists among soil physicochemical properties, microbial community structure, enzyme activities, leaf photosynthetic performance, and fruit quality.

To test these hypotheses, this study was conducted in typical ponkan orchards exhibiting significant differences in fruit quality. By comparing aboveground quality performance with belowground soil environmental characteristics, we aimed to elucidate the soil–microbe system features associated with high-quality fruit formation. The specific objectives were as follows: (1) To compare and analyze the differences in soil physicochemical properties, enzyme activities, and microbial community structures between the orchards with higher and lower comprehensive performance; (2) To employ multi-index correlation analysis to reveal the synergistic relationships among soil physicochemical properties, microbial community structure, enzyme activities, leaf photosynthetic performance, and fruit quality, and to identify the combination of soil ecological characteristics closely associated with higher comprehensive performance; (3) To propose data-driven soil management strategies based on the research findings, providing practical guidance for sustainable high-quality production in ponkan orchards. Through systematic multi-index and multi-dimensional correlation analysis, this study aimed to elucidate, from an ecological perspective, the synergistic characteristics of soil abiotic and biotic factors associated with higher comprehensive performance, thereby providing theoretical support for soil management in high-quality ponkan cultivation and contributing to the sustainable development of the ponkan industry.

## 2. Results

### 2.1. Differences in Fruit Quality and Yield of Ponkan Orchards with Different Comprehensive Performance Levels

Significant differences were observed in fruit yield and quality across different ponkan orchards (Table 1). Both the yield per plant (Yield), yield per hectare and the single fruit weight (SFW) in Orchards 3 and 4 were significantly higher than those in Orchards 1 and 2. Regarding fruit quality, with the exception of total soluble solids (TSS) content, which showed no significant difference from Orchard 3, Orchard 4 exhibited significantly higher values in soluble solids, vitamin C (Vc) content, and the TSS/TA compared to the other orchards, while its titratable acid (TA) content was the lowest among all orchards. Among the three soluble sugars, sucrose (Suc) accounted for the highest proportion, ranging from 35.79 to 58.22 mg g^−1^. There was no significant difference in sucrose content between Orchards 3 and 4, but both were significantly higher than Orchards 1 and 2. Additionally, Orchard 4 had the highest glucose (Glu) and fructose (Fru) contents, reaching 22.16 mg g^−1^ and 19.83 mg g^−1^, respectively, which were significantly greater than those in the other orchards.

### 2.2. Soil Nutrient Characteristics of the Ponkan Orchards with Different Comprehensive Performance Levels

**Figure 1:**
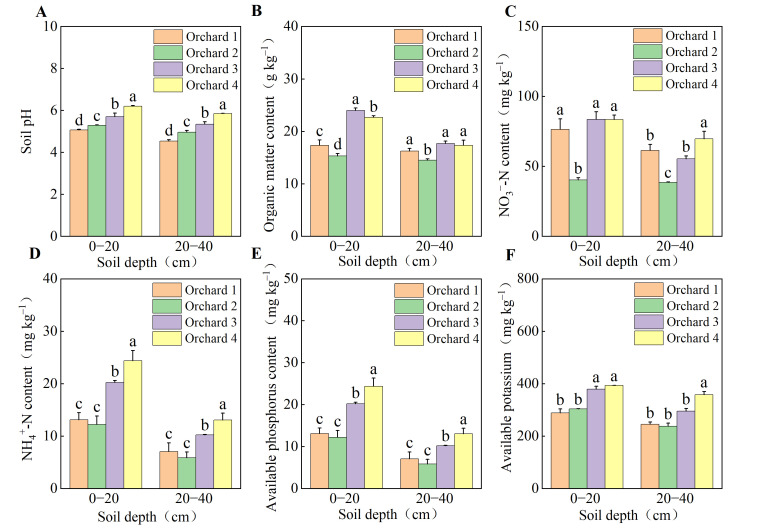
Soil pH (**A**), organic matter (**B**), NO_3_^−^-N (**C**), NH_4_^+^-N (**D**), available phosphorus (**E**) and available potassium (**F**) in the ponkan orchards with different comprehensive performance levels. NH_4_^+^-N, ammonium nitrogen; NO_3_^−^-N, nitrate nitrogen. Values followed by different letters differ significantly among the ponkan orchards with different comprehensive performance levels (*p* < 0.05); the data are expressed as means ± standard deviation (*n* = 3).

Soil nutrient characteristics differed significantly between the ponkan orchards with higher comprehensive performance and those with relatively lower comprehensive performance (Figure 1). In both the 0–20 cm and 20–40 cm soil layers, the pH in Orchard 4 was significantly higher than that in the other orchards, followed by the Orchard 3. In the 0–20 cm layer, the soil organic matter (SOM) content was 24.01 g kg^−1^ in Orchard 3 and 22.69 g kg^−1^ in Orchard 4, both being significantly higher than those in Orchard 1 and Orchard 2. The soil nutrient status in Orchard 3 and Orchard 4 was significantly better than that in Orchard 1 and Orchard 2—the former had significantly higher contents of ammonium nitrogen (NH_4_^+^-N), nitrate nitrogen (NO_3_^−^-N), available phosphorus (AP), and available potassium (AK) than the latter; moreover, the overall nutrient level in Orchard 4 was superior to that in Orchard 3.

### 2.3. Soil Enzyme Activities of the Ponkan Orchards with Different Comprehensive Performance Levels

**Figure 2:**
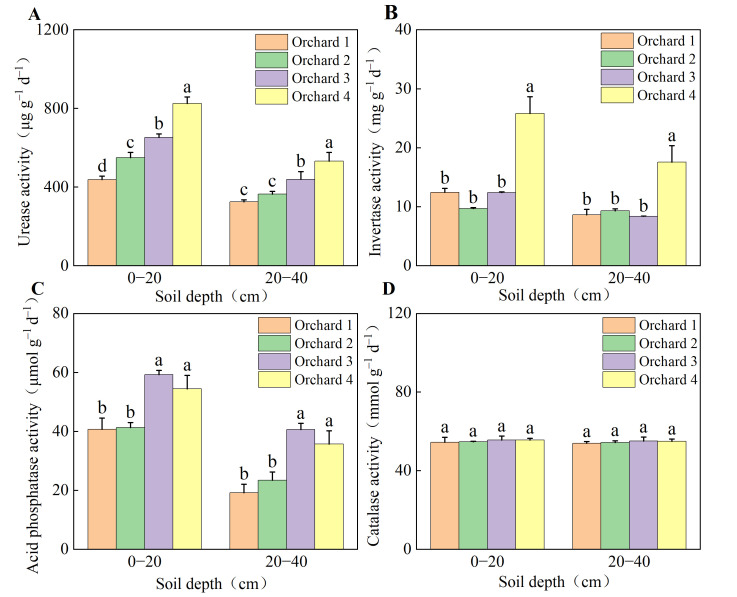
Soil urease activity (**A**), invertase activity (**B**), acid phosphatase activity (**C**), and catalase activity (**D**) in the ponkan orchards with different comprehensive performance levels. Values followed by different letters differ significantly among the ponkan orchards with different comprehensive performance levels (*p* < 0.05); the data are expressed as means ± standard deviation (*n* = 3).

Soil enzyme activities differed significantly between the ponkan orchards with higher comprehensive performance and those with relatively lower comprehensive performance (Figure 2). Regarding urease (URE) and acid phosphatase (ACP) activities, Orchard 3 and Orchard 4 exhibited significantly higher values than the other orchards in both soil layers, with the urease activity in Orchard 3 being second only to that in Orchard 4, while acid phosphatase showed no significant difference between the two. Invertase (INV) activity was highest in Orchard 4, which was significantly higher than in the other orchards. In contrast, no significant differences in Invertase activity were detected among Orchard 1, Orchard 2, and Orchard 3 in either soil layer. No significant differences were observed in soil catalase (CAT) activity across all sampling areas.

### 2.4. Characteristics of Soil Bacterial Community Richness and Diversity in the Ponkan Orchards with Different Comprehensive Performance Levels

Soil bacterial community richness and diversity differed significantly between the ponkan orchards with higher comprehensive performance and those with relatively lower comprehensive performance (Table 2). Regarding bacteria, the Chao1 index was highest in Orchard 3 for both the 0–20 cm and 20–40 cm soil layers, showing significant superiority over the other orchards. The highest Shannon index was recorded in Orchard 4 (9.79 at 0–20 cm; 9.38 at 20–40 cm). In the 0–20 cm layer, the Shannon index of Orchard 4 showed no significant difference from that of Orchard 3 but was significantly higher than that of Orchard 1 and Orchard 2. In the 20–40 cm layer, it did not differ significantly from Orchard 1 or Orchard 3 but was significantly higher than that of Orchard 3. The Simpson index reached 0.99 in both soil layers for Orchard 1, Orchard 3, and Orchard 4, which was significantly higher than the values for Orchard 2 (0.98 at 0–20 cm; 0.96 at 20–40 cm).

The characteristics of soil fungal diversity indices are presented in Table 3. Except for the Simpson index in the 0–20 cm layer, Orchard 4 exhibited the highest values for the Chao1, Shannon, and Simpson indices across both soil layers, followed by the Orchard 3. In contrast, all indices were the lowest in the Orchard 2. These results indicate that the richness and diversity of soil fungi in the Orchard 3 and Orchard 4 were relatively superior to those in the other ponkan cultivation orchards.

### 2.5. Characteristics of Soil Fungal Community Composition and Structure in the Ponkan Orchards with Different Comprehensive Performance Levels

**Figure 3:**
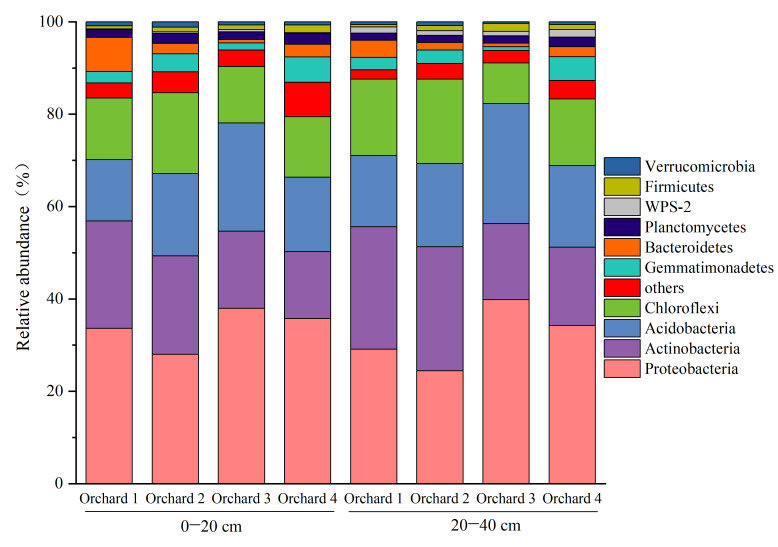
Structure of soil bacterial communities in ponkan orchards with the different comprehensive performance levels.

The relative abundance of bacterial communities at the phylum level was analyzed and presented in a bar chart (Figure 3). The dominant phyla were generally consistent across the two soil layers. In the 0–20 cm layer, the predominant bacterial phyla included Proteobacteria (27.99–37.94%), Acidobacteria (14.47–16.71%), Actinobacteria (16.11–23.43%), and Chloroflexi (12.23–13.07%). In the 20–40 cm layer, Proteobacteria (24.42–39.83%), Acidobacteria (15.38–26.02%), Actinobacteria (16.45–26.89%), and Chloroflexi (8.76–18.26%) remained dominant. Comparative analysis among different orchard areas revealed that, in both the 0–20 cm and 20–40 cm layers, the relative abundance of Proteobacteria was significantly higher in Orchard 3 and Orchard 4 than in Orchard 1 and Orchard 2. Conversely, the relative abundance of Actinobacteria was significantly lower in Orchard 3 and Orchard 4 compared to Orchard 1 and Orchard 2.

**Figure 4:**
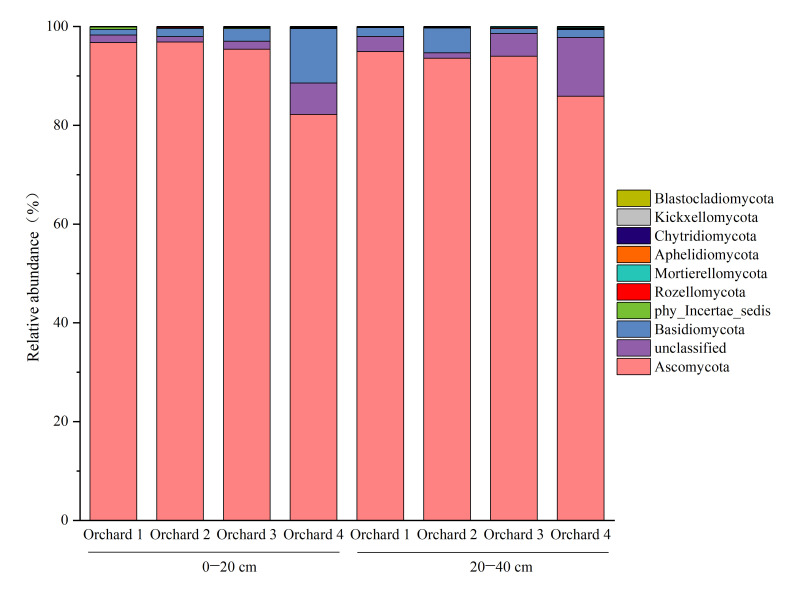
Structure of soil fungal communities in the ponkan orchards with different comprehensive performance levels.

Ascomycota was absolutely dominant in the 0–20 cm soil layer, and its relative abundance in Orchard 4 was significantly lower than that in the other orchards. In the 20–40 cm soil layer, Ascomycota remained the dominant phylum, with relative abundance ranging from 82.18% to 96.83%, and no significant differences were observed among the orchards. The relative abundance of Basidiomycota ranged from 1.01% to 1.57% in Orchard 3 and Orchard 4, while it ranged from 1.79% to 5.00% in Orchard 2 and Orchard 1. The abundances of the remaining fungal phyla also remained extremely low (Figure 4).

### 2.6. Leaf Photosynthetic Characteristics in the Ponkan Orchards with Different Comprehensive Performance Levels

**Figure 5:**
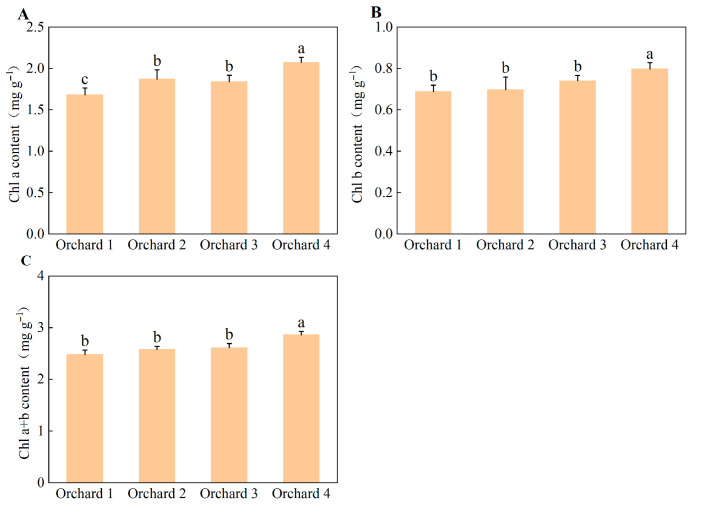
Leaf chlorophyll a (**A**), chlorophyll b (**B**) and chlorophyll a+b (**C**) content in the ponkan orchards with different comprehensive performance levels. Values followed by different letters differ significantly among the ponkan orchards with different comprehensive performance levels (*p* < 0.05); the data are expressed as means ± standard deviation (*n* = 3).

Significant differences in leaf chlorophyll content were observed among the ponkan orchards with different comprehensive performance levels (Figure 5). In Orchard 4, chlorophyll a, chlorophyll b, and total chlorophyll content in ponkan leaves were all significantly higher than in the other three areas.

**Figure 6:**
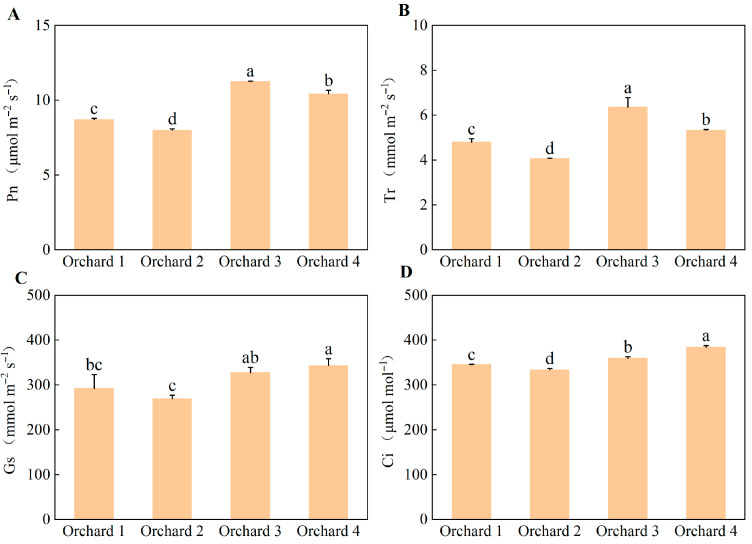
Leaf Pn (**A**), Tr (**B**), Gs (**C**), and Ci (**D**) in the ponkan orchards with different comprehensive performance levels. Pn, net photosynthetic rate; Tr, transpiration rate; Gs, stomatal conductance; Ci, intercellular CO_2_ concentration. Values followed by different letters differ significantly among the ponkan orchards with different comprehensive performance levels (*p* < 0.05); the data are expressed as means ± standard deviation (*n* = 3).

Photosynthetic gas exchange parameters among the ponkan orchards with different comprehensive performance levels are shown in Figure 6. Except for the absence of a significant difference in stomatal conductance between Orchard 3 and Orchard 1, the net photosynthetic rate (Pn), transpiration rate (Tr), stomatal conductance (Gs), and intercellular CO_2_ concentration (Ci) were all significantly higher in the Orchard 3 and Orchard 4 than in the Orchard 1 and Orchard 2. Specifically, Pn was highest in Orchard 3, which was 40.78% and 29.22% higher than in Orchard 2, and Orchard 1, respectively.

**Figure 7:**
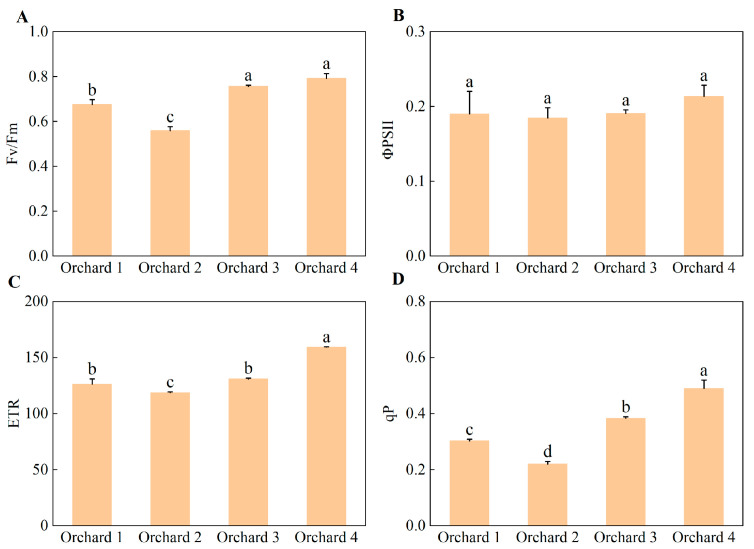
Leaf Fv/Fm (**A**), ΦPSII (**B**), ETR (**C**), and qP (**D**) in the ponkan orchards with different comprehensive performance levels. Fv/Fm, maximum photochemical efficiency; qP, photochemical quenching coefficient; ΦPSII, actual photochemical efficiency; ETR, electron transport rate. Values followed by different letters differ significantly among the ponkan orchards with different comprehensive performance levels (*p* < 0.05); the data are expressed as means ± standard deviation (*n* = 3).

Chlorophyll fluorescence parameters in the ponkan orchards with different comprehensive performance levels are shown in Figure 7. The maximum photochemical efficiency (Fv/Fm) and photochemical quenching coefficient (qP) were significantly higher in the Orchard 3 and Orchard 4 than in the Orchard 1 and Orchard 2. No significant differences were observed in the actual photochemical efficiency (ΦPSII) among the areas. The electron transport rate (ETR) was highest in the Orchard 4 and significantly higher than in the other three areas; there was no significant difference in ETR between Orchard 1 and Orchard 3, and both were significantly higher than that in Orchard 2.

**Figure 8:**
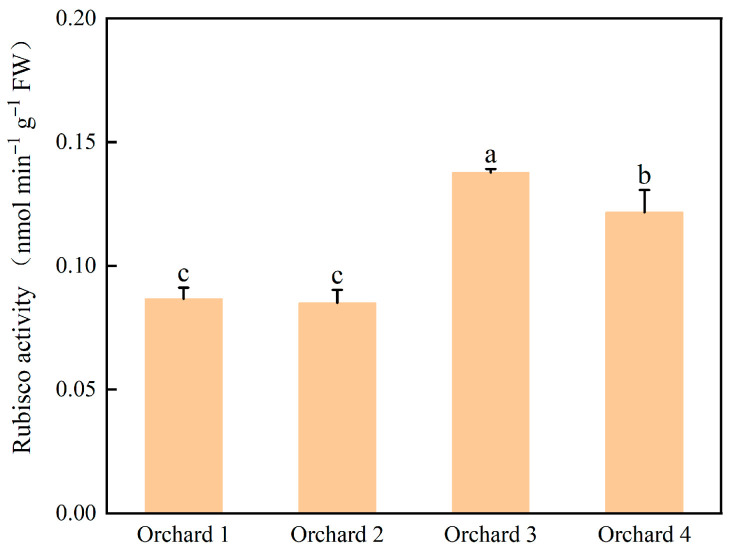
Rubisco activities in the ponkan orchards with different comprehensive performance levels. Rubisco, ribulose-1,5-bisphosphate carboxylase/oxygenase. Values followed by different letters differ significantly among the ponkan orchards with different comprehensive performance levels (*p* < 0.05); the data are expressed as means ± standard deviation (*n* = 3).

Leaf Rubisco activities in the ponkan orchards with different comprehensive performance levels are shown in Figure 8. Rubisco activity was highest in the Orchard 3, at 0.137 nmol min^−1^ g^−1^, significantly higher than in Orchards 1, 2, and 4 by 58.84%, 61.96%, and 7.83%, respectively.

### 2.7. Correlation Analysis

**Figure 9:**
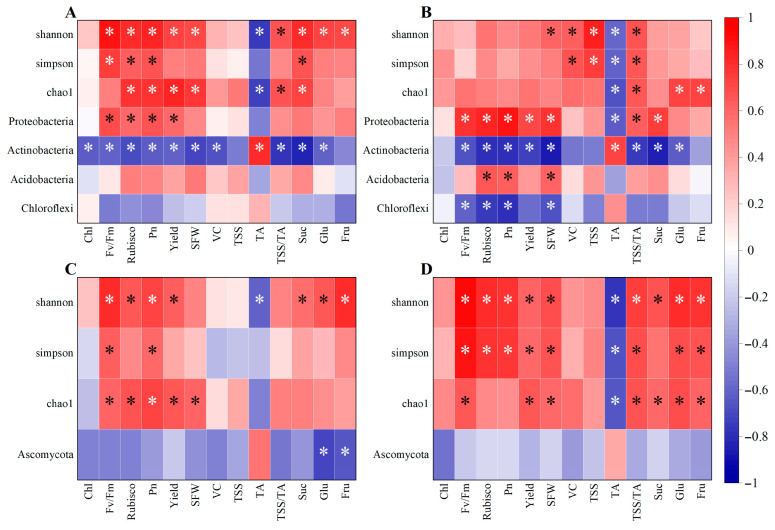
Pearson correlation analysis between soil microbial community structure and leaf photosynthetic parameters as well as fruit quality in ponkan orchards. Correlation between bacterial diversity indices and relative abundance of dominant bacterial phyla with leaf photosynthetic parameters and fruit quality at 0–20 cm depth (**A**), correlation between bacterial diversity indices and relative abundance of dominant bacterial phyla with leaf photosynthetic parameters and fruit quality at 20–40 cm depth (**B**), correlation between fungal diversity indices and relative abundance of dominant fungal phyla with leaf photosynthetic parameters and fruit quality at 0–20 cm depth (**C**), correlation between fungal diversity indices and relative abundance of dominant fungal phyla with leaf photosynthetic parameters and fruit quality at 20–40 cm depth (**D**), * indicates *p* < 0.05.

Pearson correlation analysis was used to examine the relationships among various soil properties, soil α-diversity indices, relative abundances of dominant microbial phyla, plant traits, and fruit quality indicators (Figure 9). The results indicated that in ponkan orchards, improving soil nutrients, soil enzyme activities, and soil alpha diversity, increasing the relative abundances of Proteobacteria and Acidobacteria, while reducing the relative abundances of Actinobacteria, Chloroflexi, and Ascomycota, could enhance leaf photosynthetic performance and fruit quality.

In the 0–20 cm soil layer, soil nutrient content, soil enzyme activities, and the Shannon, Simpson, and Chao1 indices of both fungal and bacterial communities were significantly positively correlated with photosynthetic parameters and fruit quality indicators. In this layer, with the exception of CAT, which showed no significant correlation with photosynthetic parameters or fruit quality, increases in the other soil nutrient contents and enzyme activities enhanced leaf photosynthesis and fruit quality in ponkan. URE and INV activities were significantly positively correlated with Suc, Glu, and Fru contents in fruit, and ACP activity was significantly positively correlated with Glu concentration. Soil pH, NH_4_^+^-N, SOM, AP, AK, URE, and ACP were significantly positively correlated with fruit yield (Figure S1A). Figure 9A,C showed that in this layer, the relative abundance of Proteobacteria was significantly positively correlated with Fv/Fm, Rubisco activity, Pn, and Yield. The relative abundance of Actinobacteria was significantly negatively correlated with photosynthetic performance and fruit quality. The relative abundance of the fungal phylum Ascomycota was significantly negatively correlated with Glu and Fru content.

In the 20–40 cm soil layer, soil nutrient content, soil enzyme activities, and the Shannon, Simpson, and Chao1 indices of fungal and bacterial communities were also significantly positively correlated with photosynthetic parameters and fruit quality indicators. In this layer, only CAT exhibited no significant correlation with either photosynthetic parameters or fruit quality. By contrast, elevated levels of other soil nutrients and enzyme activities generally improved leaf photosynthesis and fruit quality in ponkan. Specifically, URE activity showed a significantly positive correlation with fruit Suc, Glu, and Fru contents; ACP activity was significantly positively correlated with Glu concentration; and INV activity was significantly and positively associated with fruit Glu and Fru contents. Meanwhile, soil pH, organic matter, available phosphorus, available potassium, URE, and ACP all displayed significantly positive correlations with fruit yield (Figure S1B). Figure 9B,D showed that in this layer, the relative abundance of Proteobacteria was significantly positively correlated with Fv/Fm, Rubisco activity, Pn, Yield, SFW, TSS/TA ratio, and Suc content, and significantly negatively correlated with TA content. Actinobacteria remained significantly negatively correlated with most of the above indicators. Acidobacteria was significantly positively correlated with Rubisco activity, Pn, and SFW. Chloroflexi was significantly negatively correlated with Fv/Fm, Rubisco activity, Pn, and SFW.

## 3. Discussion

### 3.1. Effects of Soil Properties on the Quality and Yield of Ponkan

This study indicates that soil properties exert a critical influence on the yield and quality of ponkan. High-quality, high-yielding orchards (Orchard 3, Orchard 4) are generally associated with more favorable soil environmental conditions. This finding aligns with previous research on peach orchards, which demonstrated that orchards with superior soil conditions exhibit higher organic matter content and nutrient availability, leading to improved fruit quality and enhanced resistance to physiological disorders [22]. It further supports the widely accepted view in studies of citrus and other fruit trees that soil pH and nutrient status play a vital role in tree growth and productivity [20,23]. Notably, an optimal soil pH not only enhances nutrient solubility and bioavailability but may also indirectly create a more favorable rhizosphere environment for root nutrient uptake by regulating the activity of soil microbial communities [24,25].

This superior soil environment directly enhanced the activity of key soil enzymes, including urease, acid phosphatase, and sucrase. Soil enzymes serve as sensitive biological indicators of soil health, as they catalyze critical processes in organic matter decomposition and nutrient cycling [26]. Notably, Orchard 4 exhibited significantly higher sucrase activity in both the 0–20 cm and 20–40 cm soil layers, which showed a direct positive correlation with higher sucrose, glucose, and fructose concentrations in the fruits (Figure S1). This positive correlation suggests that soil organic matter transformation may be more active in high-yield orchards, which could theoretically help sustain nutrient supply, further support the photosynthetic function of trees, and ultimately promote sugar accumulation.

### 3.2. Effects of Bacterial Communities on Ponkan Quality and Yield

Our analysis of the microbial community revealed that soil biological characteristics may serve as important factors underlying fruit quality in ponkan orchards. The orchards with high yield and quality exhibited greater bacterial richness and diversity, as reflected by the Chao1 and Shannon indices, and these diversity metrics were positively correlated with fruit soluble sugar content. This finding aligns well with the biodiversity-ecosystem function (BEF) hypothesis, which proposes that higher microbial diversity contributes to ecosystem stability and promotes nutrient cycling efficiency [27,28]. It is also worth noting that three major phyla, namely Proteobacteria, Acidobacteria, and Actinobacteria, together accounted for over 80% of the total sequences across all samples. This observation aligns with the notion that citrus orchard soils may harbor a relatively conserved core microbial community [29].

Proteobacteria, known for their broad metabolic diversity, showed a significant positive correlation with key leaf photosynthetic parameters, fruit quality indicators, and soil fertility indices. This is consistent with its recognized role as a dominant phylum in fertile agricultural soils, primarily due to its versatile carbon utilization capacity [30]. The enrichment of Proteobacteria may promote plant growth through two main pathways: first, by enhancing organic matter mineralization and nutrient cycling, such as nitrogen transformation, thereby directly improving nutrient bioavailability [31]; second, by encompassing numerous plant growth-promoting rhizobacteria that can directly stimulate root development and function through the production of plant hormones, phosphorus solubilization, or pathogen inhibition [32]. Studies have shown that agricultural management can reshape soil microbial communities by altering soil aggregate properties and promote the growth of aerobic heterotrophic microorganisms, which supports our conclusion that the favorable soil conditions in high-performance orchards foster beneficial microbial groups such as Proteobacteria [33].

Notably, in contrast to previous findings on pomelo, the ecological role of Actinobacteria in the present study exhibited a markedly different pattern. While a previous study reported that the relative abundance of Actinobacteria was positively correlated with soil pH, organic matter, and yield per plant in pomelo orchards [34]. This phylum showed significant negative correlations with most photosynthetic parameters, fruit quality traits, and yield in our study. Accordingly, its relative abundance in the topsoil of high-quality orchards was significantly lower than that in low-quality orchards. This discrepancy can be explained by the ecological strategy of Actinobacteria. As a phylum typically favored in oligotrophic or stressed environments [30,35]. Its lower abundance in nutrient-rich soils may indirectly reflect the favorable soil nutrient status and low-stress conditions characteristic of high-yielding orchards. The contrasting findings across studies suggest that the ecological function of Actinobacteria is not fixed but rather highly dependent on specific soil contexts and crop species.

Similarly, the negative correlation of Chloroflexi in the subsoil suggests that its oligotrophic niche preference is incompatible with the active nutrient cycling required for high yield. Chloroflexi is typically more abundant in nutrient-poor subsoils and primarily metabolizes recalcitrant organic matter [36], which may explain its negative association with high productivity in this system. In contrast, Acidobacteria in the subsoil was positively correlated with key physiological and yield parameters. This indicates a beneficial role in maintaining subsoil carbon–nitrogen balance and supporting deep root health. Acidobacteria is involved in the decomposition of complex organic matter and is more adapted to environments with a diverse range of carbon sources [37]. Our results further support the important functional role of Acidobacteria in subsoil ecosystems, particularly in sustaining deep root growth and nutrient uptake, which is crucial for high citrus yield and quality. These findings are consistent with previous studies conducted in citrus orchards, in which higher microbial diversity and the enrichment of nutrient—cycling microorganisms (e.g., nitrogen-fixing bacteria, phosphate-solubilizing bacteria) have been identified as key characteristics of healthy orchard soils. Our results, together with those previous studies, highlight the importance of functional microbial communities in improving soil fertility and fruit quality in perennial fruit tree systems [38]. It should be noted that these proposed mechanisms are inferences based on the existing literature rather than direct experimental evidence from the present study. Further targeted experiments, such as strain isolation and inoculation assays, are still required to verify these hypothesized pathways.

### 3.3. Effects of Soil Fungal Communities on Ponkan Quality and Yield

The diversity of fungal communities (Shannon index) was also positively correlated with fruit sugar accumulation. However, structural analysis revealed more complex relationships, which is consistent with previous studies emphasizing the diverse and environment-dependent roles of fungi in agricultural ecosystems. Although Ascomycota dominated all samples (relative abundance > 80%), its relative abundance in the topsoil was significantly negatively correlated with fruit glucose and fructose contents. This may be attributed to the potential pressure from pathogenic fungi and nutrient competition in the soil. Certain phytopathogenic Ascomycetes present in the soil can infect plant roots even without causing obvious aboveground diseases, which may consume plant photosynthates or trigger plant defense responses, thus diverting carbohydrate resources otherwise allocated to fruit development; they may also inhibit root function, indirectly impair the absorption of water and nutrients, and ultimately reduce photosynthetic efficiency and sugar accumulation [39,40]. In addition, some highly saprophytic Ascomycetes may compete with plant roots for available nitrogen, phosphorus and other nutrients in the soil during the decomposition of organic matter, and such competition can limit the nutrient acquisition of fruit trees, especially under conditions where nutrient supply is not extremely sufficient [41].

## 4. Materials and Methods

### 4.1. Experimental Orchards

The experimental sites were the Tianma and Hongweichang Ponkan Bases, located in Yongchun County, Fujian Province, China (25°40′ N, 118°29′ E and 25°17′ N, 118°18′ E, respectively). The study area belongs to a subtropical monsoon climate zone, with a mean annual temperature of 21.2 °C and annual precipitation averaging 1813.7 mm. The soil used in this study was classified as Ultisol (USDA Soil Taxonomy) and Acrisol (WRB) based on its development from Quaternary red clay parent material under subtropical monsoon climate, with characteristics of acidic pH, low base saturation, and subsurface clay accumulation.

Based on a preliminary survey conducted in 2021 of multiple orchards in two large ponkan production bases, Tianma and Hongweichang, in Yongchun County, the experimental orchards were selected according to the following criteria: (1) Orchards were located in typical planting bases of the main production area, with consistent soil type, tree age, rootstock, and historical field management practices; (2) Based on comprehensive performance of fruit quality and yield, two types of orchards with significant differences were selected, namely; lower-performance orchards and higher-performance orchards. According to the above criteria, a total of four representative orchards were selected and designated as Orchard 1, Orchard 2, Orchard 3, and Orchard 4. Orchards 2 and 3 were located in the Tianma Ponkan Base, while Orchards 1 and 4 were located in the Hongweichang Ponkan Base. Orchards 1 and 2 exhibited lower comprehensive performance in fruit quality and yield, whereas Orchards 3 and 4 performed favorably. All experimental orchards comprised 10-year-old ponkan trees grafted onto Poncirus trifoliata rootstock, with a planting density of approximately 900 trees ha^−1^ and drip irrigation management. The fertilization regime was consistent across all orchards: 1 kg compound fertilizer (17.00% N, 17.00% P_2_O_5_, 17.00% K_2_O) per tree in February and July; 1 kg compound fertilizer (13.00% N, 6.00% P_2_O5, 32.00% K_2_O) per tree in October; 5 kg of commercial fermented chicken manure organic fertilizer per tree in December. The organic fertilizer was produced by Fujian Lvtun Biotechnology Co., Ltd. (Nanping, China), with a nutrient content of 2.36% N, 3.17% P_2_O_5_, 12.03% K_2_O, and 40.5% organic matter.

### 4.2. Sample Collection

Soil samples were collected on 5 July 2022. In each experimental orchard, 9 uniform, healthy fruit trees without disease or pest symptoms were randomly selected. Within the drip line of each tree, soil samples were collected from two soil layers (0–20 cm and 20–40 cm) using a diagonal sampling method. The 9 trees were randomly divided into three groups (3 trees per group). Soil samples from the same orchard, same soil layer, and same group were thoroughly mixed to form one composite sample as a biological replicate. A total of three biological replicates (three independent composite samples) were established for each soil layer in each orchard. After removing stones and plant debris on-site, the samples were placed in an insulated container with ice packs and transported to the laboratory on the same day. The samples were then processed for different analyses: one portion was stored at 4 °C for the determination of ammonium nitrogen, nitrate nitrogen content, and soil enzyme activity; another portion was stored at −80 °C for soil DNA extraction and high-throughput sequencing; and a third portion was air-dried, passed through a 1 mm sieve, and used to measure pH, available phosphorus, available potassium, and organic matter.

Leaf samples were collected on 15 July 2022. In each experimental orchard, the same trees used for soil sampling were selected, and the 9 trees were divided into three groups (three trees per group) following the grouping scheme for soil sampling. Healthy, mature, fully expanded functional leaves were collected from the southeastern outer periphery of each tree crown. These leaves were used for the determination of chlorophyll fluorescence, photosynthetic parameters, chlorophyll content, and Rubisco activity. Chlorophyll fluorescence and photosynthetic parameters were measured first, after which the leaves were immediately placed in a 4 °C ice box and transported to the laboratory for subsequent analysis of chlorophyll content and Rubisco enzyme activity.

Fruit samples were collected at the maturity stage on 30 November 2022 (214 days after full bloom), with three biological replicates. Each replicate consisted of 27 fruits, totaling 81 fruits per orchard. A portion of the fruits was used for fruit quality determination, while the remaining portion was flash-frozen in liquid nitrogen and stored at −80 °C for the analysis of soluble sugar content in the pulp.

### 4.3. Determination of Soil Chemical Properties and Enzyme Activities

Soil pH was measured using a pH meter (PHS-3C, Leici, Shanghai, China) with a 1:2.5 (soil:water, w:v) suspension method [21]. Soil organic matter (SOM) was determined by the potassium dichromate oxidation method [21]. The contents of soil ammonium nitrogen, nitrate nitrogen, available potassium, and available phosphorus were measured using commercial assay kits (Solarbio, Beijing, China) [42]. Activities of soil urease, catalase, invertase, and acid phosphatase were also determined using commercial assay kits (Solarbio, Beijing, China) [43].

### 4.4. Determination of Leaf Photosynthetic Physiology and Biochemical Indicators

Healthy, mature functional leaves with consistent growth, orientation, and leaf position were selected. Under standardized conditions, approximately 400 µmol mol^−1^ CO_2_, 1000 µmol m^−2^ s^−1^ photosynthetic photon flux density (PPFD), leaf temperature 26.1 ± 0.3 °C, and relative humidity 60 ± 3%, leaves were allowed to acclimate to the chamber conditions for 10 min until steady-state photosynthesis was achieved. Subsequently, net photosynthetic rate, intercellular CO_2_ concentration, stomatal conductance, and transpiration rate were measured on 14 July 2022, between 8:30 and 11:00 AM using two CIRAS-4 portable photosynthesis and fluorescence system (PP Systems, Amesbury, MA, USA). Three replicate measurements were performed on each leaf, and the average value was calculated. Five leaves were selected from each plant, with the mean value serving as the representative value for the individual plant. Three plants were set for each treatment as biological replicates (*n* = 3).

For the determination of chlorophyll fluorescence parameters, leaves were subjected to a 30 min dark adaptation at ambient orchard temperature with a leaf clip before measurement. Measurements were conducted between 8:30 and 11:00 AM on 15 July 2022, using the same leaves as those for gas exchange measurements. Subsequently, a chlorophyll fluorescence imaging system (IMAGING-PAM, Heinz Walz GmbH, Effeltrich, Germany) was used to determine the maximum photochemical efficiency (Fv/Fm), actual photochemical efficiency of PSII (ΦPSII), apparent electron transport rate (ETR), and photochemical quenching coefficient (qP). Chlorophyll and carotenoid contents were extracted with 95% (*v*/*v*) ethanol solution under dark conditions and measured using a spectrophotometer [44]. Rubisco enzyme activity was measured following the instructions of the ribulose-1,5-bisphosphate carboxylase/oxygenase assay kit (Solarbio, Beijing, China).

### 4.5. Determination of Fruit Quality

Individual fruit weight was measured using an electronic scale with an accuracy of 0.01 g. Six segments were taken from each fruit, weighed, and recorded. The segments were then mixed and juiced using a juice extractor, with the process repeated three times. The extracted juice was used to determine vitamin C (Vc) content, total soluble solids (TSS) content, titratable acid (TA) content, and the TSS/TA ratio [45]. Vc content was measured using the 2,6-dichloroindophenol (DIP) reduction method; TSS content was determined using a handheld digital refractometer; TA content was measured by titration with 0.1 mol L^−1^ NaOH, and the TSS/TA ratio was calculated accordingly.

The contents of sucrose, glucose, and fructose were determined following the method [46,47]. Specifically, 0.5 g of fruit flesh was ground in liquid nitrogen, mixed with 6 mL of deionized water, and extracted by ultrasonication at 40 °C for 40 min. After cooling to room temperature, the mixture was centrifuged at 8000× *g* for 10 min at 4 °C, and the entire supernatant was collected. An appropriate volume of the supernatant was aspirated using a 1 mL syringe, filtered through a 0.22 μm aqueous filter membrane, and analyzed using a high-performance liquid chromatography (HPLC) system. The chromatographic conditions were as follows: mobile phase consisting of acetonitrile and 0.2% ammonia water (88:12, *v*/*v*), an NH_2_ column, injection volume of 2 μL, flow rate of 0.3 mL min^−1^, column temperature of 35 °C, detection cell temperature of 80 °C, and injection duration of 20 min.

### 4.6. Soil DNA Extraction, Amplification, and Sequencing

Genomic DNA was extracted from soil samples using the SDS method, and its purity and concentration were determined. According to the selection of sequencing regions, specific primers with Barcode and high-fidelity DNA polymerase were used for PCR amplification of the V3–V4 variable region of the bacterial 16S rRNA gene and the fungal ITS1 region (primers ITS1F/ITS2).

PCR products were verified by 2% agarose gel electrophoresis, and target bands were excised and recovered using the AxyPrep DNA Gel Extraction Kit (Axygen Biosciences, Union City, CA, USA). Based on the preliminary quantitative results of electrophoresis, the recovered products were accurately quantified using the QuantiFluor™-ST Blue Fluorometric System (Promega Corporation, Madison, WI, USA). Samples were mixed in equimolar ratios according to sequencing requirements, and high-throughput sequencing was performed on the Illumina NovaSeq 6000 platform by Shanghai Applied Protein Technology Co., Ltd. to construct small-fragment libraries for paired-end sequencing.

Raw sequencing data were spliced using FLASH v1.2.11 and quality-controlled and filtered using QIIME 1.9.1. After quality control, bacterial samples yielded an average of 70,138 valid reads per sample, and fungal samples yielded an average of 49,124 reads per sample. The UPARSE algorithm was used to cluster sequences into operational taxonomic units (OTUs) at a 97% similarity threshold, and chimeras were removed. Representative sequences were annotated for species using the RDP classifier based on the SILVA database (for bacteria) and the UNITE database (for fungi), with a confidence threshold of 0.7.

Rarefaction was performed based on the minimum sequencing depth to ensure comparability among samples. The Good’s coverage index of each sample was 0.98–0.99 (for bacteria) and 0.98–1.00 (for fungi), all greater than 0.97, indicating sufficient sequencing coverage.

### 4.7. Biodiversity Indices

The Chao1, Shannon, and Simpson indices were used to evaluate the diversity and richness of soil microbial communities. The Chao1 index estimates the total species richness of the community, while the Shannon and Simpson indices reflect community diversity and evenness. The above-mentioned α-diversity indices were calculated from the rarefied OTU table using QIIME 1.9.1 [48].

### 4.8. Statistic Analysis

Data were processed using Microsoft Excel 2010 (Microsoft Inc., Redmond, WA, USA) to calculate means and standard deviations. Statistical analyses were performed in SPSS 26.0 (International Business Machines Corporation, New York, NY, USA). The Shapiro–Wilk test and Levene’s test were used to assess normality and homogeneity of variances, respectively. After confirming homogeneity of variances, one-way analysis of variance (ANOVA) was conducted, and Duncan’s multiple range test was applied at a significance level of *p* < 0.05 to compare differences among treatment means. All graphs were generated using Origin 2021 (OriginLab Corp., Northampton, MA, USA), with error bars representing ± standard deviation. Pearson correlation coefficients were used to explore the relationships among variables. Since P-values were not adjusted for multiple testing, the results should be interpreted cautiously as exploratory analysis. We focused on correlations with strong effect sizes (|r| > 0.6).

## 5. Conclusions

This study demonstrates that variations in the productivity and fruit quality of ponkan orchards are rooted in soil–microbe synergistic effects. Orchards with higher comprehensive performance exhibited soil pH values closer to the optimal range (5.5–6.5), along with greater nutrient availability, stronger enzyme activities, and a more optimized microbial community structure. These orchards displayed a distinct microbial distribution pattern: Proteobacteria dominated in the topsoil, while Acidobacteria were enriched in the subsoil, both of which were positively correlated with leaf photosynthetic efficiency and fruit sugar accumulation. Conversely, the dominance of Actinobacteria in the topsoil, and the enrichment of Chloroflexi and certain Ascomycota in the subsoil, indicated suboptimal soil conditions and were negatively correlated with fruit quality formation.

These findings provide practical insights for orchard management in high-quality ponkan production: regulating soil pH to the optimal range through the judicious application of soil amendments can effectively enhance nutrient availability and microbial activity; meanwhile, adopting agronomic practices that improve the soil microecological environment can help promote beneficial microorganisms while suppressing potentially detrimental ones.

Future research could focus on the following directions: (1) Mechanistic validation—integrating metagenomics and culturomics to isolate key strains within Proteobacteria and Acidobacteria, and validating their regulatory pathways using models such as SEM/RDA; (2) Causal validation—inoculating candidate strains into low-fertility soils and confirming their growth-promoting effects on fruit quality through field or pot experiments; (3) Technology development—validating the generalizability of research findings across multiple orchard systems and developing composite microbial fertilizers based on native key strains. The ultimate goal is to establish a replicable and scalable cultivation system based on the integrated regulation of soil–microbe interactions, thereby providing scientific and technological support for high-quality ponkan production.

## Figures and Tables

**Table 1:** Differences in fruit quality and yield among the different ponkan orchards.

Quality Index of Ponkan	Orchard 1	Orchard 2	Orchard 3	Orchard 4
yield per plant (kg)	31.65 ± 1.35 b	32.44 ± 0.43 b	37.54 ± 1.59 a	37.01 ± 1.95 a
yield per hectare (t ha^−1^)	28.49 ± 1.22 b	29.20 ± 0.39 b	33.79 ± 1.43 a	33.31 ± 1.76 a
SFW (g)	134.07 ± 4.27 c	140.25 ± 2.64 b	162.40 ± 1.43 a	160.50 ± 1.47 a
Vc content (mg 100g^−1^)	27.81 ± 0.50 c	36.54 ± 0.50 b	35.24 ± 1.33 b	41.71 ± 0.87 a
TSS content (%)	10.47 ± 0.37 c	11.83 ± 0.36 b	12.70 ± 0.45 a	12.70 ± 0.09 a
TA content (%)	1.20 ± 0.01 a	0.86 ± 0.02 c	0.90 ± 0.04 b	0.68 ± 0.01 d
TSS/TA	8.75 ± 0.35 d	13.75 ± 0.10 c	14.11 ± 0.09 b	18.72 ± 0.18 a
Suc content (mg g^−1^ FW)	35.79 ± 5.50 b	36.88 ± 5.60 b	54.81 ± 5.69 a	58.22 ± 6.06 a
Glu content (mg g^−1^ FW)	15.45 ± 1.00 bc	14.94 ± 1.67 c	17.94 ± 1.24 b	22.16 ± 1.04 a
Fru content (mg g^−1^ FW)	16.78 ± 1.17 b	15.45 ± 1.61 b	17.28 ± 0.61 b	19.83 ± 0.53 a

SFW, single fruit weight; Vc, vitamin C; TSS, total soluble solids; TA, titratable acidity; Suc, sucrose; Glu, glucose; Fru, fructose. Values followed by different letters differ significantly among the different ponkan orchards (*p* < 0.05); the data are expressed as means ± standard deviation (*n* = 3).

**Table 2:** Soil bacterial diversity indices in the ponkan orchards with different comprehensive performance levels.

Soil Layers	Orchards	Chao1 Index	Shannon Index	Simpson Index
0–20 cm	Orchard 1	3260.71 ± 378.38 c	8.81 ± 0.34 b	0.99 ± 0.01 a
	Orchard 2	3673.9 ± 340.68 bc	7.91 ± 0.48 c	0.98 ± 0.01 b
	Orchard 3	5052.80 ± 272.31 a	9.63 ± 0.20 a	0.99 ± 0.01 a
	Orchard 4	4227.14 ± 107.60 b	9.79 ± 0.22 a	0.99 ± 0.01 a
20–40 cm	Orchard 1	3365.04 ± 490.99 c	8.84 ± 0.51 a	0.99 ± 0.01 a
	Orchard 2	3506.56 ± 389.37 c	7.26 ± 0.36 b	0.96 ± 0.02 b
	Orchard 3	4619.98 ± 105.85 a	8.98 ± 0.22 a	0.99 ± 0.01 a
	Orchard 4	4115.06 ± 288.99 b	9.38 ± 0.34 a	0.99 ± 0.01 a

Values followed by different letters differ significantly among the ponkan orchards with different comprehensive performance levels (*p* < 0.05); the data are expressed as means ± standard deviation (*n* = 3).

**Table 3:** Soil fungal diversity indices in the ponkan orchards with different comprehensive performance levels.

Soil Layers	Orchards	Chao1 Index	Shannon Index	Simpson Index
0–20 cm	Orchard 1	125.71 ± 3.51 b	4.02 ± 0.33 a	0.88 ± 0.03 a
	Orchard 2	162.57 ± 19.62 ab	3.25 ± 0.50 b	0.77 ± 0.04 b
	Orchard 3	184.68 ± 31.64 ab	4.36 ± 0.31 a	0.90 ± 0.04 a
	Orchard 4	237.22 ± 20.30 a	4.46 ± 0.21 a	0.89 ± 0.01 a
20–40 cm	Orchard 1	116.20 ± 9.84b	3.31 ± 0.17 bc	0.75 ± 0.02 bc
	Orchard 2	88.66 ± 20.20b	2.40 ± 0.39 c	0.63 ± 0.05 c
	Orchard 3	134.80 ± 30.19b	4.27 ± 0.21 ab	0.86 ± 0.01 ab
	Orchard 4	235.89 ± 28.61a	4.98 ± 0.24 a	0.91 ± 0.04 a

Values followed by different letters differ significantly among the ponkan orchards with different comprehensive performance levels (*p* < 0.05); the data are expressed as means ± standard deviation (*n* = 3).

## Data Availability

Data are contained within the article. The data presented in this study can be requested from the authors.

## Supplementary Materials

The following supporting information can be downloaded at: https://www.mdpi.com/article/10.3390/plants15050819/s1, Figure S1. Pearson correlation analysis of soil nutrients and enzyme activities with leaf photosynthetic parameters and fruit quality in ponkan orchards.
