# Supplementary material for: Impacts of Soil Properties and Microbial Community on Fruit Quality and Yield in Ponkan Orchards with Different Comprehensive Performance
Source: Plants (Basel). 2026 Mar 7;15(5):819. doi: 10.3390/plants15050819 (PMC12987050; doi:10.3390/plants15050819)
Supplement: Supplementary file 1 [file plants-15-00819-s001.zip › plants-4164373-supplementary.pdf]

plants

MDPI

Supplementary Material

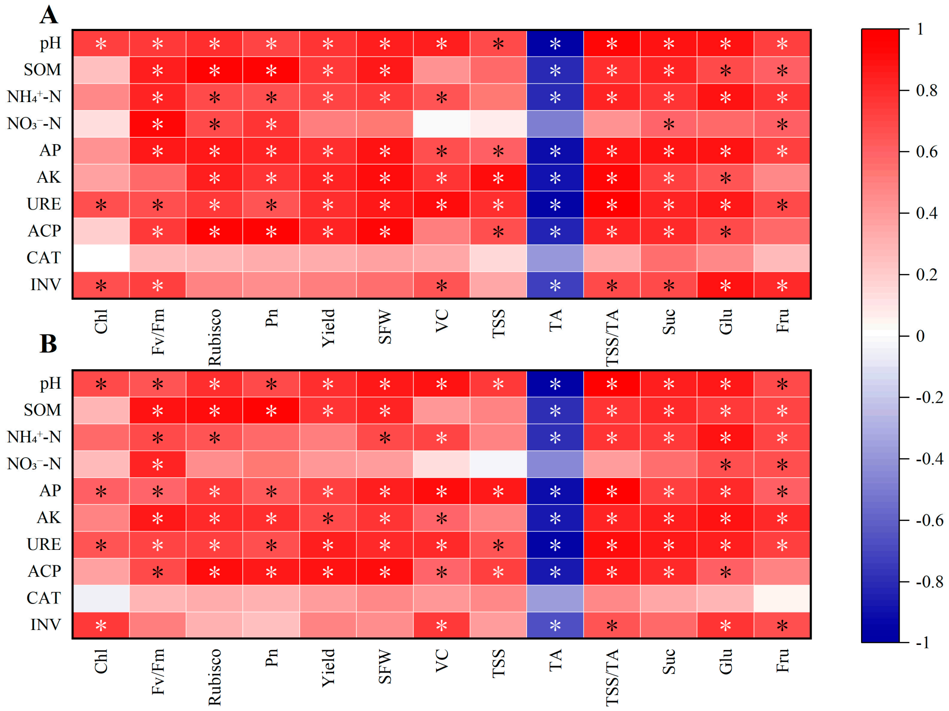

Figure S1. Pearson correlation analysis of soil nutrients and enzyme activities with leaf photosynthetic parameters and fruit quality in ponkan orchards. Correlation between soil nutrients and enzyme activities with leaf photosynthetic parameters and fruit quality at 0–20 cm depth (**A**), Correlation between soil nutrients and enzyme activities with leaf photosynthetic parameters and fruit quality at 20–40 cm depth (**B**). * indicates $p < 0.05$.
